# CircHIF1A induces cetuximab resistance in colorectal cancer by promoting HIF1α-mediated glycometabolism alteration

**DOI:** 10.1186/s13062-024-00478-x

**Published:** 2024-05-07

**Authors:** Yiting Geng, Xiao Zheng, Dachuan Zhang, Shanshan Wei, Jun Feng, Wei Wang, Luo Zhang, Changping Wu, Wenwei Hu

**Affiliations:** 1 Department of Oncology, The Third Affiliated Hospital of Soochow University, Changzhou, 213003 Jiangsu China; 2 Department of Tumor Biological Treatment, The Third Affiliated Hospital of Soochow University, Changzhou, 213003 Jiangsu China; 3 Jiangsu Engineering Research Center for Tumor Immunotherapy, The Third Affiliated Hospital of Soochow University, Changzhou, 213003 Jiangsu China; 4 Department of Pathology, The Third Affiliated Hospital of Soochow University, Changzhou, 213003 Jiangsu China

**Keywords:** Colorectal cancer, Cetuximab, CircHIF1A, HIF1α

## Abstract

Epidermal growth factor receptor (EGFR)-targeted therapy is an important treatment for RAS wild-type metastatic colorectal cancer (mCRC), but the resistance mechanism remains unclear. Here, the differential expression of circRNAs between Cetuximab sensitive and resistant cell lines was analyzed using whole-transcriptome sequencing. We identified that the expression of circHIF1A was significantly higher in LIM1215-R than in LIM1215. When treated with Cetuximab, downregulation of circHIF1A level weakened the proliferation and clonal formation ability of LIM1215-R, caused more cells to enter G0-G1 phase, and significantly reduced the basal respiration, ATP production, and maximal respiration, as well as the glycolytic capacity and glycolytic reserve. The response rate and prognosis of circHIF1A-positive patients were inferior to those of negative patients. Mechanistically, circHIF1A can upregulate the level of hypoxia-inducible factor 1 A (HIF1A) by competitively binding to miR-361-5p, inducing the overexpression of enzymes such as glucose transporter 1 (GLUT1) and lactate dehydrogenase A (LDHA). In a xenograft model, inhibition of circHIF1A expression increased the sensitivity to Cetuximab treatment. In conclusion, circHIF1A can promote HIF1α-mediated glycometabolism alteration to induce Cetuximab resistance in CRC. It has the potential to become a screening indicator for the Cetuximab beneficial population in mCRC and a new therapeutic target for enhancing treatment efficacy.

**Supplementary Information:**

The online version contains supplementary material available at 10.1186/s13062-024-00478-x.

## Background

Colorectal cancer (CRC) is one of the most prevalent and lethal malignancies globally [1, 2]. Some patients exhibit metastasis at the time of diagnosis, making them ineligible for surgical treatment. The leading causes of death are postoperative recurrence and distant metastasis, which remain major challenges in comprehensive treatment of CRC.

Metastatic colorectal cancer (mCRC) primarily relies on medicine therapy to alleviate symptoms and prolong survival. Over the past few decades, with the development and combined use of chemotherapy drugs like 5-Fluorouracil (5-FU), Oxaliplatin, and Irinotecan, the prognosis of mCRC patients has improved to some extent. Since the beginning of the 21st century, epidermal growth factor receptor (EGFR)-targeted monoclonal antibodies (mAbs), such as Cetuximab and Panitumumab, have become breakthroughs in the treatment of mCRC. Combined with chemotherapy, these mAbs result in a median overall survival (OS) of over 31 months for RAS wild-type mCRC patients [3], significantly improving the quality of life and prognosis of patients. The status of the RAS gene (wild/mutant type) is an effective molecular predictor of sensitivity to anti-EGFR therapy [4, 5]. However, approximately 35–40% of RAS wild-type mCRC patients do not achieve complete or partial response after treatment with Cetuximab, and sensitive patients will develop resistance after an average of 10-11.4 months [6–8]. For CRC patients with potentially resectable liver and/or lung metastases, guidelines including the National Comprehensive Cancer Network (NCCN) and the European Society for Medical Oncology (ESMO) recommend conversion therapy to achieve curative surgery. In clinical practice, due to a lack of further precise screening criteria beyond the RAS gene mutation status, some RAS wild-type mCRC patients who do not respond to anti-EGFR therapy may lose the opportunity of R0 resection after progressive disease (PD). Therefore, primary and acquired resistance are important factors limiting the application of anti-EGFR therapy for mCRC, but the specific mechanisms are not yet clear and may involve abnormal expression or activation of multiple molecules and signaling pathways, including genetic alterations such as mutations in BRAF and PIK3CA, amplification of human epidermal growth factor receptor-2 (HER-2) and MET, PTEN deletion, as well as genome hypermethylation [4, 9, 10]. There is little research on non-genetic mechanisms of mCRC resistance to anti-EGFR mAbs. Hence, it is necessary to investigate the molecular mechanisms that contribute to resistance against anti-EGFR therapy in RAS wild-type mCRC, search for biomarkers to accurately screen beneficial patients, and develop effective strategies to overcome resistance.

Circular RNA (circRNA) plays a rich biological function in malignant tumors progression [11–13]. Some circRNAs are significantly dysregulated in CRC tissue, and most of them act as miRNA sponges that exert regulatory control over key processes such as proliferation, invasion, and metastasis of CRC cells. They are associated with tumor size, degree of differentiation, lymph node metastasis, clinical staging [14, 15], and even KRAS mutation [16]. Some of them can serve as prognostic markers for patients with CRC [14, 17]. Recently, it has been found that dysregulated expression of circRNAs in CRC, breast cancer and non-small cell lung cancer (NSCLC) can lead to resistance to chemotherapy or targeted therapy [18–21], and resistance can be reversed by intervening with specific circRNAs expression in tumor cells [22]. In our previous review [23], some non-coding RNAs (ncRNAs) can influence the malignant phenotype of CRC by regulating gene expression or the activity of certain signaling pathways such as RAS/RAF/MEK/MAPK or PI3K/AKT, thereby mediating anti-EGFR mAbs resistance [17, 24, 25]. Presently, research in this field predominantly centers around microRNAs (miRNAs) and long non-coding RNAs (lncRNAs), which requires further exploration. This study identified differences in circRNA expression profiles between Cetuximab sensitive and resistant CRC cell lines using whole-transcriptome sequencing, further screened circRNAs that can affect sensitivity to Cetuximab treatment and explored the molecular mechanisms. It may provide a theoretical basis for circRNA as a screening indicator for mCRC patients who benefit from anti-EGFR therapy and a new target for overcoming resistance.

## Materials and methods

### Cell culture and transfection

Human CRC cell line LIM1215 was obtained from the Shanghai Institute of Biochemistry and Cell Biology, Chinese Academy of Sciences. Cells were cultured in Dulbecco’s modified Eagle medium (DMEM) supplemented with 10% fetal bovine serum at 37 °C in a humidified atmosphere with 5% CO_2_. Cetuximab resistant cell line LIM1215-R was established by inducing stepwise increases in dosage. Hsa_circ_0007976 overexpressing plasmids, small interfering RNA (siRNA) and short hairpin RNA (shRNA) targeting hsa_circ_0007976, hypoxia-inducible factor 1 A (HIF1A) overexpression plasmids, and negative control plasmids were purchased from General Biosystems (Hefei, Anhui, China). MiRNA-361-5p mimics, miRNA-361-5p inhibitors, and negative controls were synthesized by General Biosystems (Hefei, Anhui, China). Transfection was carried out using Lipofectamine 3000 according to the manufacturer’s instructions. The sequence of each vector is listed in Supplementary Table 1.

### Patients

RAS/BRAF wild-type, microsatellite stability (MSS) mCRC patients who were confirmed as adenocarcinoma by pathology and received Cetuximab plus fluorouracil-based combination chemotherapy (FOLFOX/CapeOX or FOLFIRI) in the Third Affiliated Hospital of Soochow University between 2017 and 2022 were included. Tumor tissue samples were obtained from patients’ previous surgical resection or colonoscopic biopsy. All patients were followed up for more than one year. Progression-free survival (PFS) was defined as the time from the first administration of Cetuximab to PD or death for any reason. This study was conducted in accordance with the Helsinki Declaration (revised in 2013) and approved by the Ethics Committee of the Third Affiliated Hospital of Soochow University (Approval No. 2021-SCRICAL-158).

### Whole-transcriptome sequencing

Whole-transcriptome sequencing was performed on LIM1215 and LIM1215-R cells (Shanghai OE Biotech Co., Ltd.). The original sequencing data were quality assessed using FastQC and quality trimmed using Trimmomatic to obtain relatively accurate and effective data. BWA was used to align the data to the reference genome, and CIRI2 was used to identify circRNAs. Variable splicing analysis was performed using CIRI-AS. The origin of the circRNAs was determined using BEDtools based on its location information and known gene annotations, and RPKM formula was used to calculate the expression level of circRNA according to BSJ reads. Based on the differential analysis results, heatmaps were plotted, and cluster analysis was performed. MiRanda was used to predict the target miRNAs of circRNA. DESeq2 was used for differential analysis of transcript expression, and a network diagram was plotted based on the association between circRNA, miRNA, and mRNA. The sequencing data that underlie the findings of this study have been archived in the National Genomics Data Center (https://ngdc.cncb.ac.cn/gsa-human). The accession number is HRA004931.

### Cell and animal experiments

The detailed procedures for cell experiments including quantitative real-time polymerase chain reaction (qRT-PCR), western blotting, cell counting kit-8 (CCK8) proliferation assay, clone formation assay, cell apoptosis and cell cycle analysis, cell metabolism assay, RNase R resistance analysis, fluorescence in situ hybridization (FISH), RNA immunoprecipitation (RIP), RNA pull-down, luciferase reporter assay, immunohistochemistry (IHC) and immunofluorescence (IF), as well as animal experiments are described in Supplementary Materials and Methods. The primer sequences are shown in Supplementary Table 2. All antibodies used in this study are listed in Supplementary Tables 3, and the sequences of probes were shown in Supplementary Table 4.

### Statistical analysis

SPSS 22.0 software was used for data analysis, and GraphPad Prism 6.0 software was used for graph generation. Difference comparisons were analyzed using t-test for two groups, and ANOVA followed by Tukey’s test for multiple group comparisons. Survival curves were plotted by the Kaplan-Meier and assessed by the log-rank test. The COX proportional hazards regression model was used to calculate the hazard ratio (HR). A *P*-value < 0.05 indicates statistical significance.

## Results

### Establishment and sensitivity verification of cetuximab resistant cell line

**Fig. 1:**
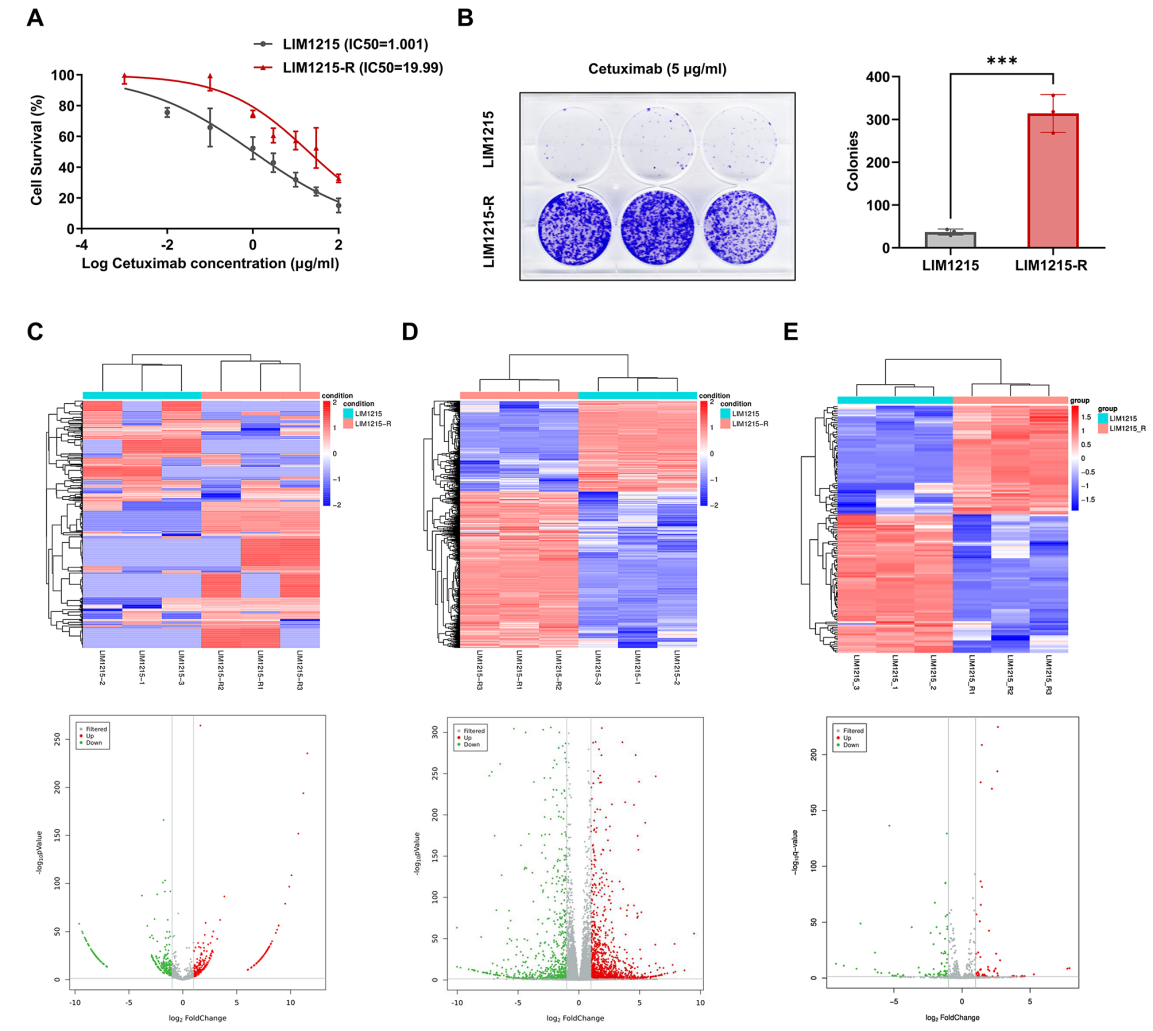
Establishment of Cetuximab resistant cell line LIM1215-R and analysis of differentially expressed RNAs. **A**-**B**. Sensitivity of LIM1215 and LIM1215-R to Cetuximab: (**A**) Cell viability of LIM1215 and LIM1215-R treated with different concentrations of Cetuximab. (**B**) Clonogenic assay (Cetuximab 5 µg/mL). C-E. Heatmaps and volcano plots of differentially expressed RNAs between LIM1215 and LIM1215-R (fold change > 2, *P* < 0.05): (**C**) circRNA expression differences; (**D**) mRNA expression differences; (**E**) miRNA expression differences. ^***^P < 0.001

The Cetuximab sensitive CRC cell line LIM1215, with RAS/BRAF wild type, was cultured and Cetuximab resistant cell line LIM1215-R was induced using a stepwise increase in dosage. The sensitivity to Cetuximab of both LIM1215 and LIM1215-R were verified (LIM1215 IC50 = 1.001, LIM1215-R IC50 = 19.99). (Fig. 1A-B). Genetic testing of LIM1215-R showed no mutations in KRAS, NRAS or BRAF (Supplementary Fig. 1).

### Analysis and verification of differentially expressed circRNAs in LIM1215 and LIM1215-R

A total of 1481 circRNAs were identified by whole-transcriptome sequencing of LIM1215 and LIM1215-R, of which 867 circRNAs were differentially expressed (fold change > 2 and *P* < 0.05), including 363 downregulated and 504 upregulated circRNAs (Fig. 1C). Furthermore, 2027 out of 16,543 mRNAs (Fig. 1D) and 141 out of 950 miRNAs (Fig. 1E) were found to be differentially expressed. Ten significantly upregulated and ten downregulated circRNAs were selected for qRT-PCR validation, as shown in Supplementary Fig. 2.

### Screening circRNAs influencing the sensitivity of CRC to cetuximab

The expression levels of the above circRNAs were respectively downregulated or overexpressed in LIM1215-R (Supplementary Fig. 3A), and the sensitivity of the corresponding cells to Cetuximab was detected. When treated with Cetuximab (5 µg/mL), among these circRNAs, only downregulation of hsa_circ_0007976 led to a significant decrease in proliferation and colony formation of LIM1215-R compared to the control group (Supplementary Fig. 3B-E), while overexpression of hsa_circ_0007976 resulted in the opposite results (Supplementary Fig. 3F-G). Without Cetuximab treatment, neither overexpression of hsa_circ_0007976 in LIM1215 nor downregulation of hsa_circ_0007976 in LIM1215-R had any effect on their proliferation or colony formation levels (Supplementary Fig. 3F-I).

**Fig. 2:**
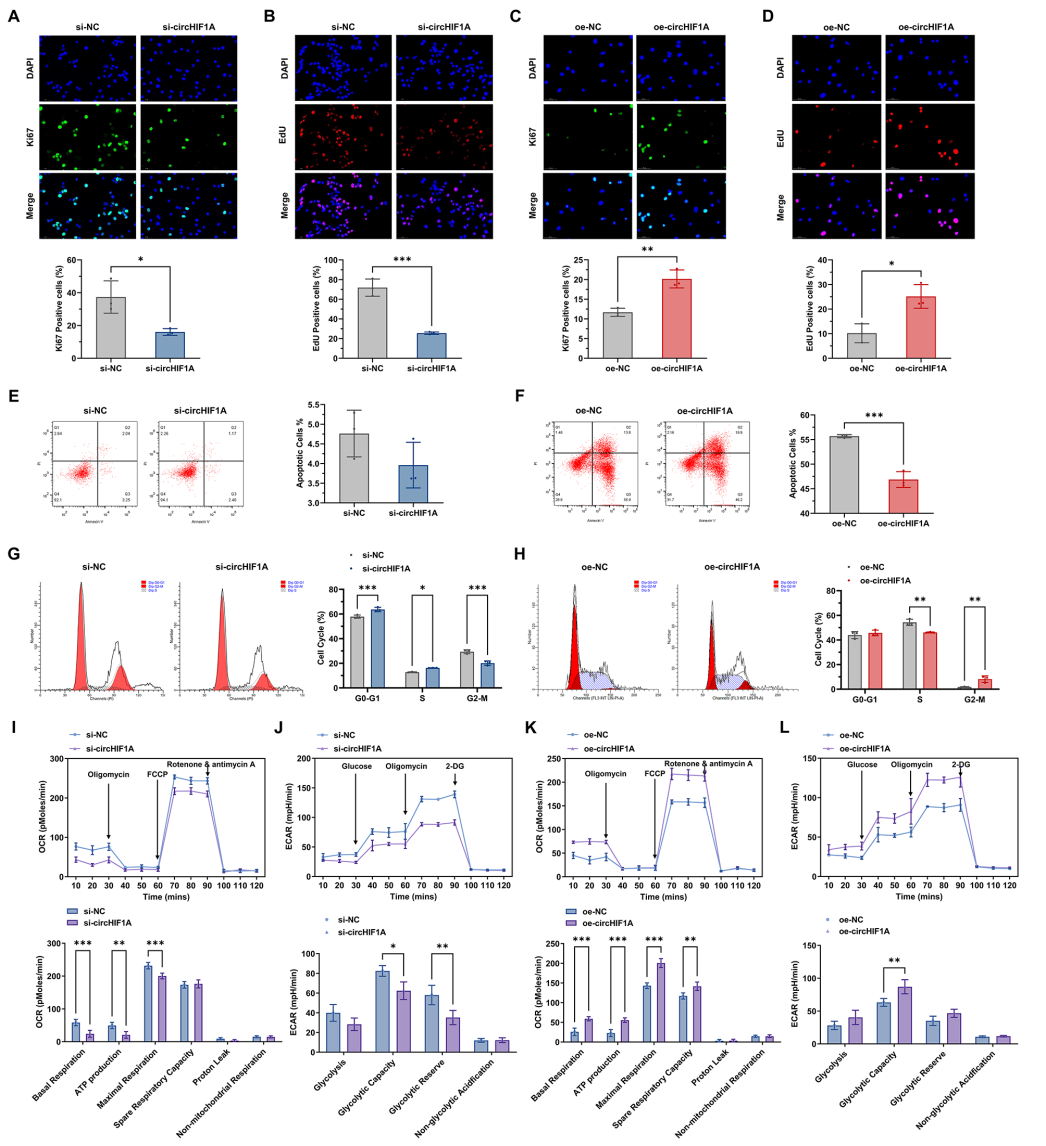
The effect of circHIF1A on CRC cell function (Cetuximab 5 µg/mL). **A**-**D**. The expression levels of Ki67 (**A**) and EdU (**B**) in LIM1215-R decreased after circHIF1A downregulation, while those in LIM1215 increased after circHIF1A overexpression (**C**-**D**). **E**-**F**. The effect of circHIF1A downregulation (**E**) and overexpression (**F**) on apoptosis levels in LIM1215-R and LIM1215, respectively, as detected by flow cytometry. **G**-**H**. The effect of circHIF1A levels on the cell cycle. **I**-**J**. Changes in aerobic metabolism (**I**) and glycolytic metabolism (**J**) in LIM1215-R after circHIF1A downregulation. **K**-**L**. Changes in aerobic metabolism (**K**) and glycolytic metabolism (**L**) in LIM1215 after circHIF1A overexpression. ^*^P < 0.05, ^**^P < 0.01, ^***^P < 0.001

Hsa_circ_0007976 is an overlapping circRNA consisting of 231 base pairs (bp), and its host gene is HIF1A located on chromosome 14. When Cetuximab was applied (5 µg/mL), downregulation of hsa_circ_0007976, also referred to as circHIF1A, led to a decrease in Ki67 (Fig. 2A) and EdU (Fig. 2B) expression levels in LIM1215-R. Conversely, overexpression of circHIF1A resulted in an increase in Ki67 (Fig. 2C) and EdU (Fig. 2D) levels in LIM1215. After downregulating circHIF1A, apoptosis level of LIM1215-R was low and not significantly different from the control group (Fig. 2E), while overexpression of circHIF1A significantly decreased apoptosis level of LIM1215 (Fig. 2F). Moreover, circHIF1A affected the cell cycle distribution, as downregulation of circHIF1A in LIM1215-R increased the proportion of cells in G0-G1 and S phase, while decreased the proportion in G2-M phase (Fig. 2G). Conversely, overexpression of circHIF1A in LIM1215 led to a decrease in S phase and an increase in G2-M phase (Fig. 2H). Additionally, circHIF1A levels also affected the aerobic metabolism and glycolysis levels in CRC cells. Compared to the control group, downregulation of circHIF1A resulted in a significant decrease in basal respiration, ATP production, and maximal respiration of LIM1215-R (Fig. 2I), as well as a significant reduction in glycolytic capacity and glycolytic reserve (Fig. 2J). Overexpression of circHIF1A in LIM1215 cells led to a significant increase in basal respiration, ATP production, maximal respiration, and spare respiratory capacity, as well as an increase in glycolytic capacity (Fig. 2K-L).

### Identification of circHIF1A and its impact on patients’ prognosis

**Fig. 3:**
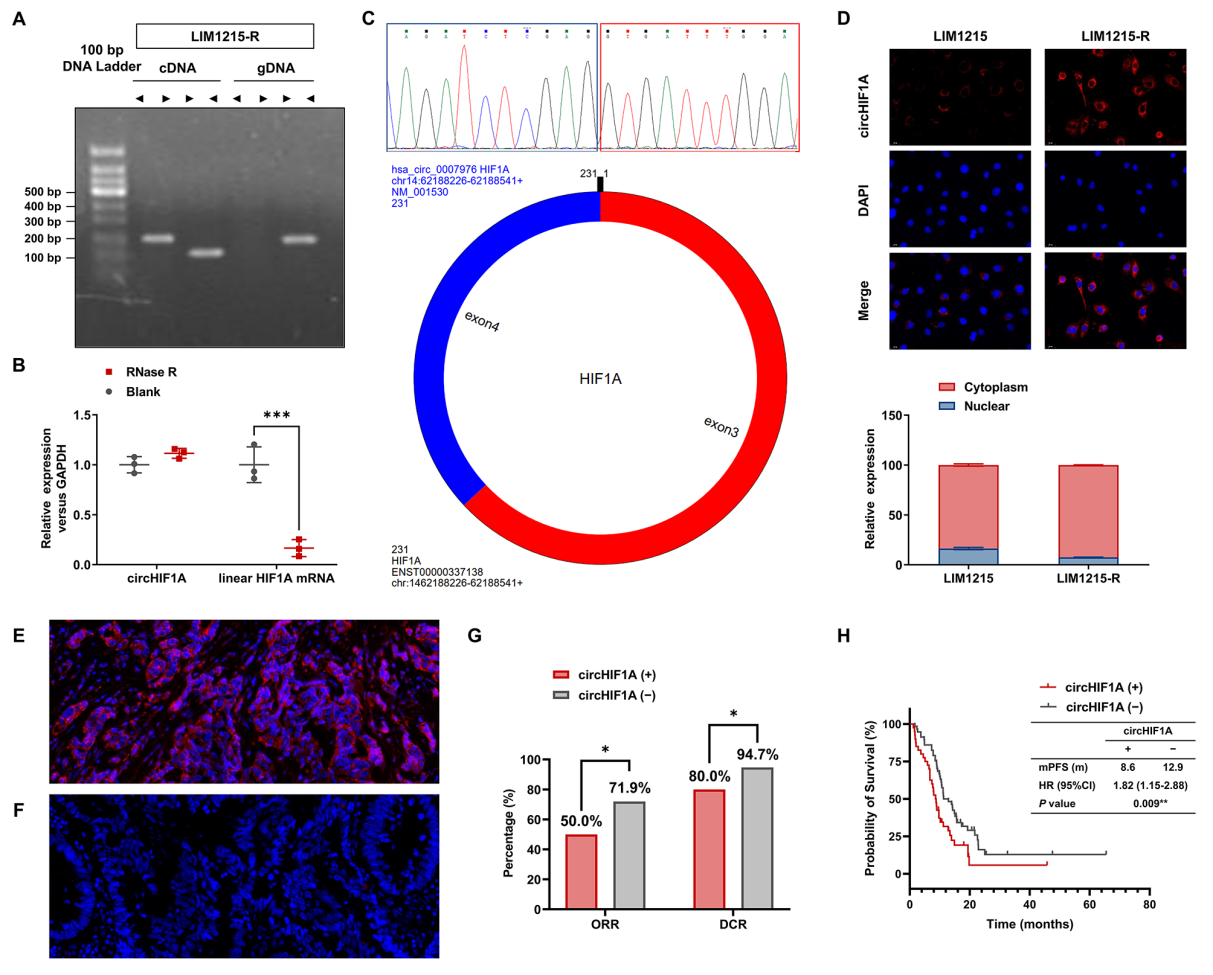
Identification of circHIF1A and its impact on patients’ prognosis. **A**-**C**. Identification of circHIF1A. **A**. qRT-PCR using divergent or convergent primers to detect circHIF1A in LIM1215-R. **B**. qRT-PCR analysis of circHIF1A and linear mRNA levels in LIM1215-R with or without RNase R treatment. **C**. Sanger sequencing showing the circular junction sequence of circHIF1A, which is formed by the splicing of the 5’ end of exon 3 and the 3’ end of exon 4. The blue box on the left represents the last 10 nucleotides of the 3’ end of exon 4, and the red box on the right represents the first 10 nucleotides of the 5’ end of exon 3. **D**. FISH detection of circHIF1A distribution in CRC cells. **E**-**F**. FISH detection of the positive (**E**) and negative (**F**) expression of circHIF1A in CRC tumor tissues (×400 magnification): the red signals represent circHIF1A labeled with Cy3 probe, and the blue signals represent the cell nuclei labeled with DAPI probe. **G**. The ORR and DCR of circHIF1A-positive patients and negative patients. **H**. The mPFS of circHIF1A-positive patients and negative patients. ^*^P < 0.05, ^**^P < 0.01, ^***^P < 0.001

Both divergent and convergent primers produced products in LIM1215-R with cDNA as template, while only the convergent primer produced a product with gDNA as template (Fig. 3A). Following treatment with RNase R, the level of HIF1A mRNA decreased significantly, whereas the level of circHIF1A remained constant (Fig. 3B). Sanger sequencing revealed that circHIF1A was a 231 bp circular splicing product formed by joining the 5’ end of exon 3 and the 3’ end of exon 4 (Fig. 3C). FISH showed that circHIF1A is primarily located in the cytoplasm, with a minority fraction present in the nucleus (Fig. 3D).

Ninety-seven mCRC patients were retrospectively selected, all of whom had wild-type RAS/BRAF genes, MSS, and were treated with Cetuximab plus fluoropyrimidine-based chemotherapy (FOLFOX/CapeOX or FOLFIRI) until PD, intolerable toxic reactions, surgery, or patient request to terminate treatment. The included patients were aged 35–82 years (median 65 years), including 75 males (77.3%) and 22 females (22.7%). Among them, 83 cases (85.6%) were left colon/rectal cancer, and 14 cases (14.4%) were right colon cancer. Diagnosed initially with advanced stage were 56 cases (57.7%) and 41 cases (42.3%) had postoperative recurrence and metastasis. Liver metastases present in 72 cases (74.2%). Eighty-one cases (83.5%) received Cetuximab as first-line treatment, while 16 cases (16.5%) received ≥ second line treatment. The reasons for discontinuing treatment among the 88/97 patients included PD (79.6%), adverse events (AE) (4.5%), surgery (5.7%), or others (10.2%). The FISH assay was performed on tumor tissue sections from patients to assess the expression of circHIF1A, with 40 cases (41.2%) being positive and 57 cases (58.8%) being negative, as shown in Fig. 3E-F. The overall objective response rate (ORR) was 62.9%, the disease control rate (DCR) was 88.7%, and the median PFS (mPFS) was 10.6 months. The ORR and DCR of circHIF1A positive patients were significantly lower than those of negative patients (ORR: 50.0% vs. 71.9%, *P* = 0.028; DCR: 80.0% vs. 94.7%, *P* = 0.024) (Fig. 3G). The mPFS of circHIF1A positive patients was also significantly inferior to that of negative patients [8.6 months vs. 12.9 months, HR (95% confidence interval, 95% CI) = 1.82 (1.15–2.88), *P* = 0.009] (Fig. 3H).

### CircHIF1A regulates the level of HIF1A through competitive binding with miR-361-5p

**Fig. 4:**
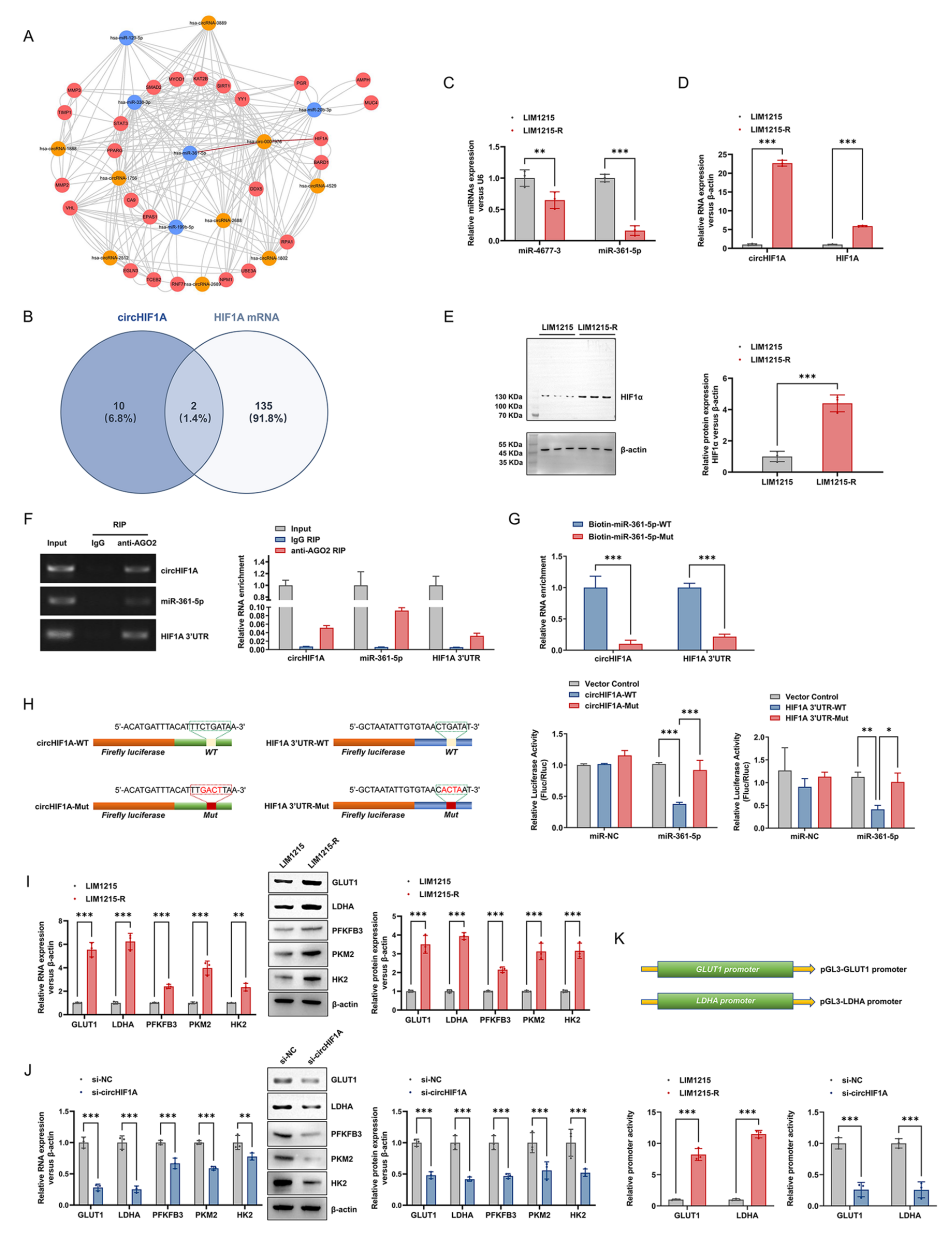
CircHIF1A/miR-361-5p/HIF1A forms a ceRNA network. **A**-**E**. Screening of target miRNAs and genes: (**A**) Cytoscape was used to generate the circRNA-miRNA-mRNA regulatory network of circHIF1A. (**B**) Target miRNAs were screened using bioinformatics methods. (**C**) Differential expression of miR-4677-3 and miR-361-5p in LIM1215 and LIM1215-R. (**D**) Differential expression of circHIF1A and HIF1A mRNA in LIM1215 and LIM1215-R. (**E**) Differential expression of HIF1α protein in LIM1215 and LIM1215-R. **F**-**H**. Validation of the binding relationship between miRNA-361-5p, circHIF1A and HIF1A: (**F**) RIP assay revealed significant enrichment of circHIF1A, miRNA-361-5p and HIF1A 3’UTR in the anti-AGO2 group compared to the IgG group. (**G**) LIM1215-R were transfected with biotin-labeled miRNA-361-5p-WT or miRNA-361-5p-Mut, and the levels of circHIF1A and HIF1A 3’UTR were analyzed by qRT-PCR. The relative ratio of IP to the input value is plotted. (**H**) Dual-luciferase reporter assays confirmed that miRNA-361-5p could bind both circHIF1A and HIF1A 3’UTR. **I**-**K**. CircHIF1A regulates downstream genes through HIF1A: (**I**) Comparison of the expression levels of GLUT1, LDHA, PFKFB3, PKM2 and HK2 mRNA and protein in LIM1215 and LIM1215-R. (**J**) Downregulation of circHIF1A in LIM1215-R resulted in altered expression of GLUT1, LDHA, PFKFB3, PKM2 and HK2 mRNA and protein. **K**. The promoter activity of GLUT1 and LDHA in LIM1215-R was higher than that in LIM1215. Downregulation of circHIF1A in LIM1215-R resulted in decreased promoter activity of GLUT1 and LDHA. ^*^P < 0.05, ^**^P < 0.01, ^***^P < 0.001

We integrated the miRDB, TargetScan, miRanda, and miRTarBase tools to predict the target genes of the first three miRNAs of circHIF1A. Then, we selected the top 15 target genes for each of the predicted circRNA-targeted miRNAs in all databases to construct a circRNA-miRNA-mRNA interaction network (Fig. 4A). Among them, the association between miR-361-5p and circHIF1A was the most prominent, and in the pool of potential target genes for miR-361-5p, HIF1A was identified as the host gene of circHIF1A. By employing the ENCORI software based on PITA, RNA22, miRMAP, microT, PicTar, TargetScan, and miRanda, miR-4677-3 and miR-361-5p were found to have potential binding relationships with circHIF1A and HIF1A (Fig. 4B). The expression of miR-4677-3 and miR-361-5p in LIM1215-R and LIM1215 exhibited a negative correlation with circHIF1A and HIF1A, and the downregulation of miR-361-5p in LIM1215-R was more significant (Fig. 4C-E).

RIP assays showed that circHIF1A, miRNA-361-5p and HIF1A 3’UTR were significantly enriched in the anti-AGO2 group compared to the IgG group (Fig. 4F), suggesting the possibility of miRNA-361-5p binding to circHIF1A and HIF1A 3’UTR, respectively. RNA pull-down experiments were conducted using biotin-labeled miR-361-5p probes in LIM1215-R, and the results demonstrated a significant enrichment of circHIF1A and HIF1A 3’UTR in the fragments captured by the wild-type miR-361-5p, as compared to the mutant-type miR-361-5p that altered the binding sites for circHIF1A or HIF1A 3’UTR (Fig. 4G). In the luciferase reporter assay, the wild- and mutant-type binding sequences of miR-361-5p for circHIF1A (circHIF1A-WT and circHIF1A-Mut) and HIF1A 3’UTR (HIF1A 3’UTR-WT and HIF1A 3’UTR-Mut) were obtained using bioinformatics methods, cloned into the psiCheck2 plasmid, and co-transfected into LIM1215-R with miR-361-5p mimic or miR-NC. Overexpression of miR-361-5p significantly decreased luciferase activity in cells transfected with circHIF1A-WT or HIF1A 3’UTR-WT plasmid, but not in cells transfected with empty vector, circHIF1A-Mut or HIF1A 3’UTR-Mut plasmid (Fig. 4H), confirming that miR-361-5p can bind to circHIF1A and HIF1A directly.

In LIM1215-R, the mRNA and protein levels of key enzymes involved in aerobic metabolism and glycolysis regulated by HIF1A, including glucose transporter 1 (GLUT1), lactate dehydrogenase A (LDHA), PFKFB3, PKM2 and HK2 were higher compared to LIM1215 (Fig. 4I), and downregulation of circHIF1A resulted in decreased expression of these enzymes (Fig. 4J). The expression alterations of GLUT1 and LDHA appeared to be the most significant. Then, pGL3-GLUT1 promoter vector and pGL3-LDHA promoter vector were constructed to detect the promoter activity of GLUT1 and LDHA. The promoter activity of GLUT1 and LDHA was significantly higher in LIM1215-R compared to LIM1215, and silencing the expression of circHIF1A in LIM1215-R using siRNA reduced the promoter activity of both GLUT1 and LDHA (Fig. 4K).

### Mir-361-5p and HIF1A can reverse circHIF1A-mediated cetuximab resistance

**Fig. 5:**
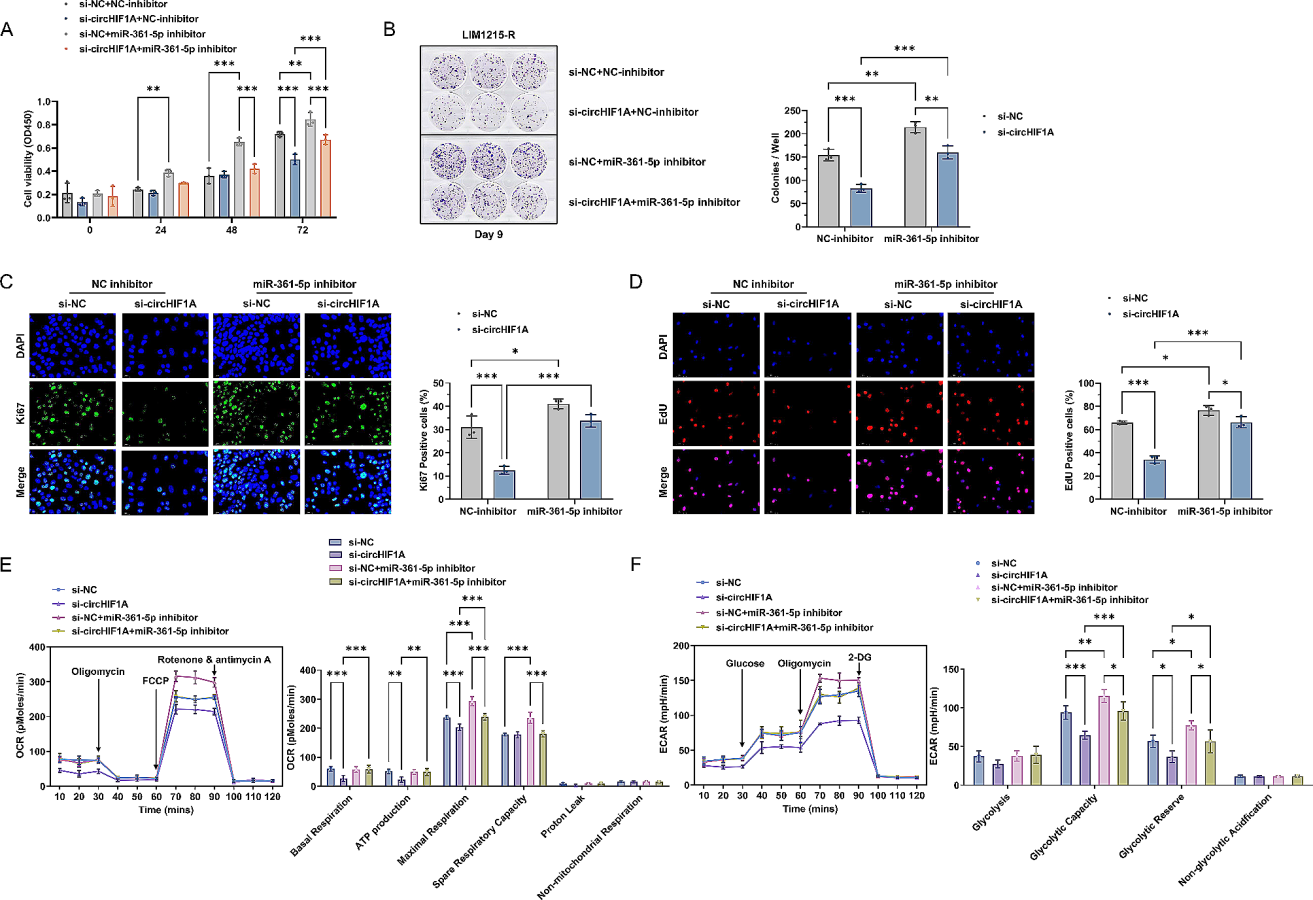
miR-361-5p intervention reverses circHIF1A-mediated Cetuximab resistance (Cetuximab 5 µg/mL). **A**-**B**. Effect of miR-361-5p downregulation on the proliferation (**A**) and colony formation (**B**) of LIM1215-R with circHIF1A downregulated. **C**-**D**. IF showed changes in the positive levels of Ki67 (**C**) and EdU (**D**) after miR-361-5p downregulation. **E**-**F**. Effect of miR-361-5p downregulation on mitochondrial metabolism (**E**) and glycolysis (**F**) in LIM1215-R. ^*^P < 0.05, ^**^P < 0.01, ^***^P < 0.001

LIM1215-R cells were treated in four groups: (1) si-NC + NC-inhibitor, (2) si-circHIF1A + NC-inhibitor, (3) si-NC + miR-361-5p inhibitor, and (4) si-circHIF1A + miR-361-5p inhibitor. The expression levels of circHIF1A, miR-361-5p, HIF1A mRNA and HIF1α protein were detected in these four groups (Supplementary Fig. 4A-D). Downregulating circHIF1A in LIM1215-R decreased the proliferation and colony formation ability, as well as the levels of Ki67 and EdU positivity, but downregulating miR-361-5p in LIM1215-R resulted in the opposite effects. In LIM1215-R with downregulated miR-361-5p, simultaneously downregulating circHIF1A, the proliferation and colony formation could not be significantly inhibited, and there were no significant changes in Ki67 and EdU positivity levels. Compared to LIM1215-R with circHIF1A downregulated alone, this group of cells had a stronger proliferation ability (Fig. 5A-D). Furthermore, in LIM1215-R with downregulated miR-361-5p and circHIF1A simultaneously, the changes in basal respiration, ATP production, maximal respiration, glycolytic capacity, and glycolytic reserve were partially reversed, which were higher than those in group 2 (Fig. 5E-F).

**Fig. 6:**
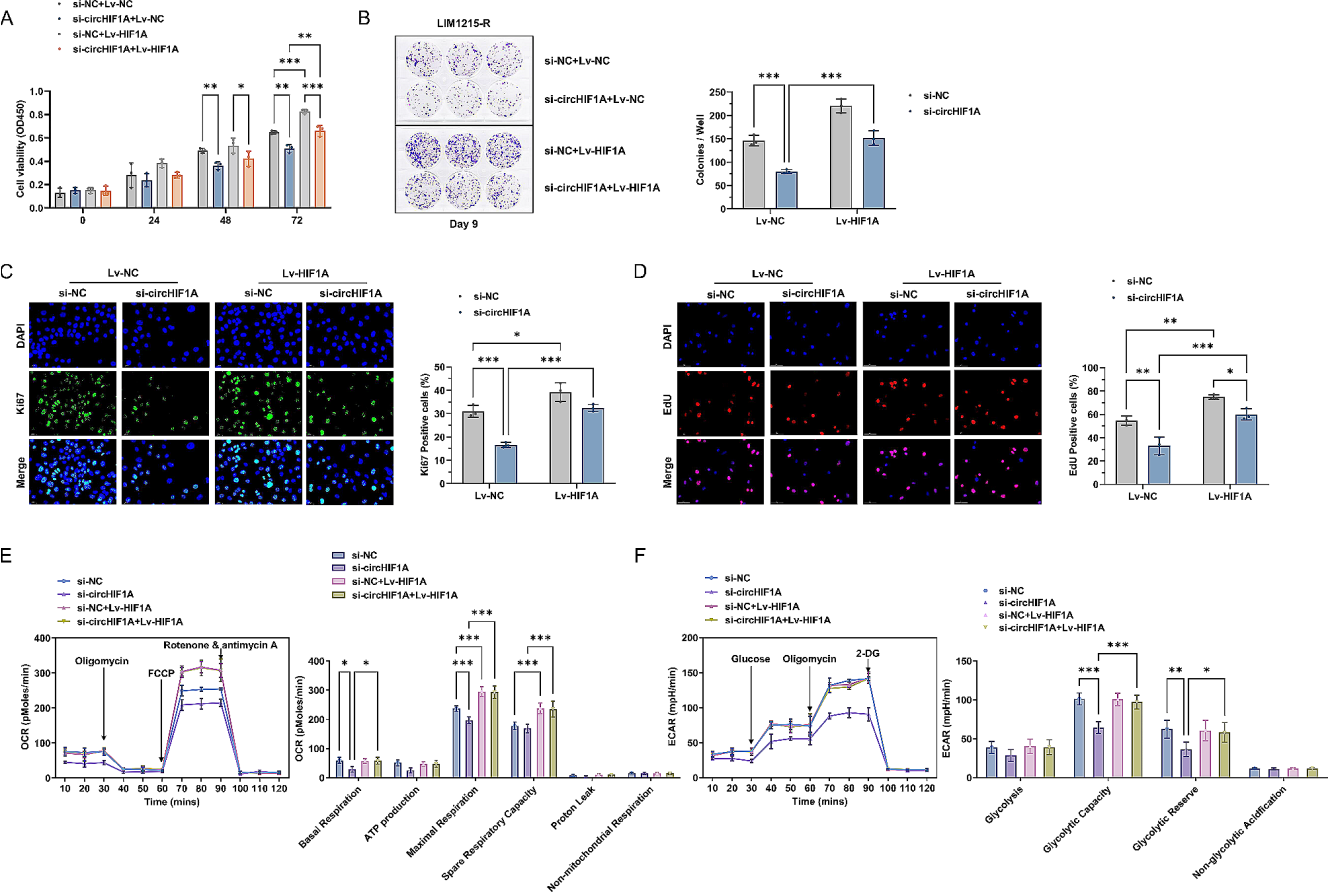
HIF1A intervention reverses circHIF1A-mediated Cetuximab resistance (Cetuximab 5 µg/mL). **A**-**B**. Effect of HIF1A overexpression on the proliferation (**A**) and colony formation (**B**) of LIM1215-R with circHIF1A downregulated. **C**-**D**. IF showed changes in the positive levels of Ki67 (**C**) and EdU (**D**) after HIF1A overexpression. **E**-**F**. Effect of HIF1A overexpression on mitochondrial metabolism (**E**) and glycolysis (**F**) in LIM1215-R. ^*^P < 0.05, ^**^P < 0.01, ^***^P < 0.001

A HIF1A overexpression vector and stable LIM1215-R transfectants were constructed, and the cells were treated in four groups: (1) si-NC + Lv-NC, (2) si-circHIF1A + Lv-NC, (3) si-NC + Lv-HIF1A, and (4) si-circHIF1A + Lv-HIF1A. The expression levels of circHIF1A, miR-361-5p, HIF1A mRNA and HIF1α protein were detected in these four groups (Supplementary Fig. 4E-H). Cellular functional experiments revealed that downregulating circHIF1A led to a decrease in proliferation and colony formation ability of LIM1215-R, as well as a decrease in Ki67 and EdU positivity levels, and overexpression of HIF1A in LIM1215-R resulted in the opposite effects. However, in LIM1215-R with HIF1A overexpression, simultaneously downregulating circHIF1A, the proliferation ability and colony formation could not be suppressed, and there were no significant decreases in Ki67 and EdU positivity levels. Compared to LIM1215-R with circHIF1A downregulated alone, this group of cells had a stronger proliferation ability (Fig. 6A-D). Furthermore, in LIM1215-R with HIF1A overexpression and circHIF1A downregulated simultaneously, the changes in basal respiration, maximal respiration, spare respiratory capacity, glycolytic capacity, and glycolytic reserve were reversed, which were higher than those in group 2 (Fig. 6E-F).

### CircHIF1A affects the sensitivity of CRC to cetuximab in an organism model

**Fig. 7:**
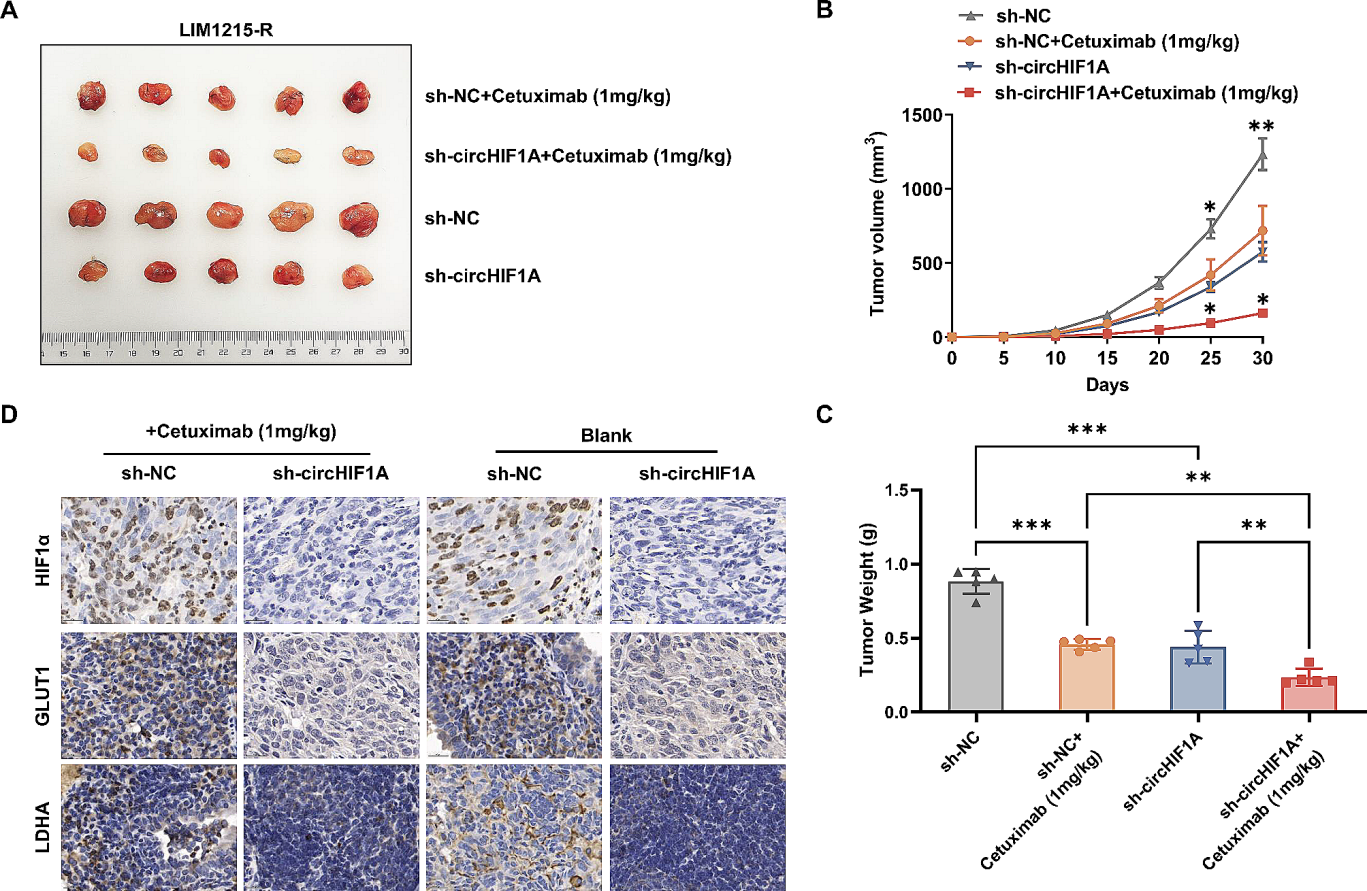
CircHIF1A affects the sensitivity of CRC to Cetuximab in an organism model. **A**. Transplanted tumors in four groups of mice. **B**-**C**. Comparison of the average volume (**B**) and weight (**C**) of tumors in the blank control group, Cetuximab control group, experimental group, and Cetuximab experimental group. **D**. IHC staining of tumor tissues showed that the expression of HIF1α, GLUT1, and LDHA in the experimental group and the Cetuximab experimental group were significantly decreased. ^*^P < 0.05, ^**^P < 0.01, ^***^P < 0.001

A mouse xenograft model was established using LIM1215-R cells. Based on the cell type injected and medicine treatment, there were four groups: (1) blank control group (LIM1215-R transfected with empty vector), (2) Cetuximab control group (LIM1215-R transfected with empty vector + Cetuximab 1 mg/kg), (3) experimental group (LIM1215-R with circHIF1A downregulated), and (4) Cetuximab experimental group (LIM1215-R with circHIF1A downregulated + Cetuximab 1 mg/kg). All mice developed tumors at the injection site. After 30 days of treatment, all the mice were euthanized. The average tumor volume and weight of the Cetuximab control group, experimental group, and Cetuximab experimental group were lower than those of the blank control group, and the Cetuximab experimental group displayed the most significant reduction in average tumor volume and weight (Fig. 7A-C). Then the tumor tissues were processed for IHC staining. In comparison to the blank control group and Cetuximab control group, the expression levels of HIF1α, GLUT1 and LDHA in the experimental group and Cetuximab experimental group were significantly reduced (Fig. 7D).

## Discussion

CRC ranks as the third most prevalent cancer worldwide and is the second leading cause of cancer-related mortality [1]. Due to limited treatment options, the 5-year survival rate of mCRC is less than 10%. Patients who have not undergone liver metastasis surgery usually survive for less than 18 months [26]. For mCRC patients who are evaluated as potentially resectable by multi-disciplinary treatment (MDT) consultations, if R0 resection of the primary and metastatic lesions can be achieved after conversion therapy, the long-term prognosis of patients can be greatly improved. Currently, Cetuximab has been established as the standard of the first-line treatment of RAS wild-type mCRC, especially for left-sided mCRC patients [6, 27]. Due to its advantages of higher ORR, early tumor regression rate (ETS), and deepness of response (DpR) [7], chemotherapy combined with Cetuximab is recommended for mCRC patients who aim for tumor reduction and surgery. However, the inability to achieve no evidence of disease (NED) postoperative due to primary resistance, or PD caused by acquired resistance, are the main reasons for treatment failure and poor prognosis. Therefore, exploring the mechanism of Cetuximab resistance is crucial for improving the survival of mCRC patients.

Studies have revealed that resistance to Cetuximab is correlated with mutations in RAS, BRAF, PIK3CA, and PTEN deletion [28]. Approximately 40.4% of mCRC patients have RAS gene mutations, with KRAS mutations accounting for 37.2% and NRAS accounting for 3.1%, which are the most important predictive biomarkers for Cetuximab sensitivity [29]. 5-7% of mCRC have BRAF V600E mutation and do not benefit from anti-EGFR mAbs treatment [27, 30]. In addition, PTEN deletion and PIK3CA mutation, HER2/HER3 pathway activation, MET amplification, upregulation of EGFR ligands and receptors, ubiquitination, rearrangement and variants of EGFR, modification of EGFR by SRC family kinases, and transactivation of alternative pathways bypassing EGFR are also involved in the resistance of Cetuximab [31–34]. However, approximately 25% of RAS, BRAF, PIK3CA, and PTEN wild-type mCRC patients exhibit resistance to Cetuximab, the underlying mechanism of which remains unclear and ncRNAs may be involved. We have previously conducted a detailed review of the research progress on ncRNAs related to anti-EGFR mAbs resistance [23]. In terms of circRNAs, circHIPK3 overexpressed in both CRC cells and tissues. As a competing endogenous RNA (ceRNA), it upregulates the expression of oncogenes FAK, IGF1R, EGFR, and YY1 by adsorbing miR-7. Suppressing the expression of circHIPK3 can increase the responsiveness of CRC cells to Cetuximab [17]. The abnormal expression of CDR1as and circRNA_0000392 in CRC regulates certain receptors or pathways associated with anti-EGFR resistance [35, 36].

In this study, circHIF1A expression was found to significantly affect the sensitivity of KRAS/NRAS/BRAF wild-type CRC cells to Cetuximab. When circHIF1A was downregulated in LIM1215-R with Cetuximab treatment, the cell proliferation was reduced, more cells entered G0-G1 phase, and the basal respiration, ATP production, maximal respiration, glycolytic capacity, and glycolytic reserve were significantly decreased. Overexpression of circHIF1A in LIM1215 significantly promoted the proliferation, reduced the apoptosis, increased the number of cells in G2-M phase, and significantly enhanced aerobic metabolism and glycolytic capacity. Besides, high level of circHIF1A also reduced the sensitivity of tumors to Cetuximab in vivo. Inhibition of circHIF1A slowed down tumor growth and improved its response to Cetuximab. According to these results, circHIF1A acts as an oncogene in CRC, and its high expression will lead to resistance of CRC to Cetuximab. CircHIF1A is a stable and non-degradable circular structure that is distributed in the cytoplasm and can be detected by FISH. In our retrospective analysis of clinical data, compared with circHIF1A-negative patients, circHIF1A-positive patients had a lower tumor regression rate and a poorer long-term prognosis. The expression level of circHIF1A in tumor tissues may be a predictive indicator for the efficacy and prognosis of RAS/BRAF wild-type mCRC patients receiving Cetuximab treatment.

In the interaction network involving circRNA-miRNA-mRNA based on the sequencing results, the relationship between miR-361-5p and circHIF1A appears to be the most significant. Interestingly, among the potential target genes regulated by miR-361-5p, HIF1A is the host gene of circHIF1A. “circHIF1A/miR-361-5p/HIF1A” formed a ceRNA feedback network, which affected the glycometabolism of CRC cells, and miR-361-5p and HIF1A intervention could reverse circHIF1A-mediated resistance to Cetuximab.

**Fig. 8:**
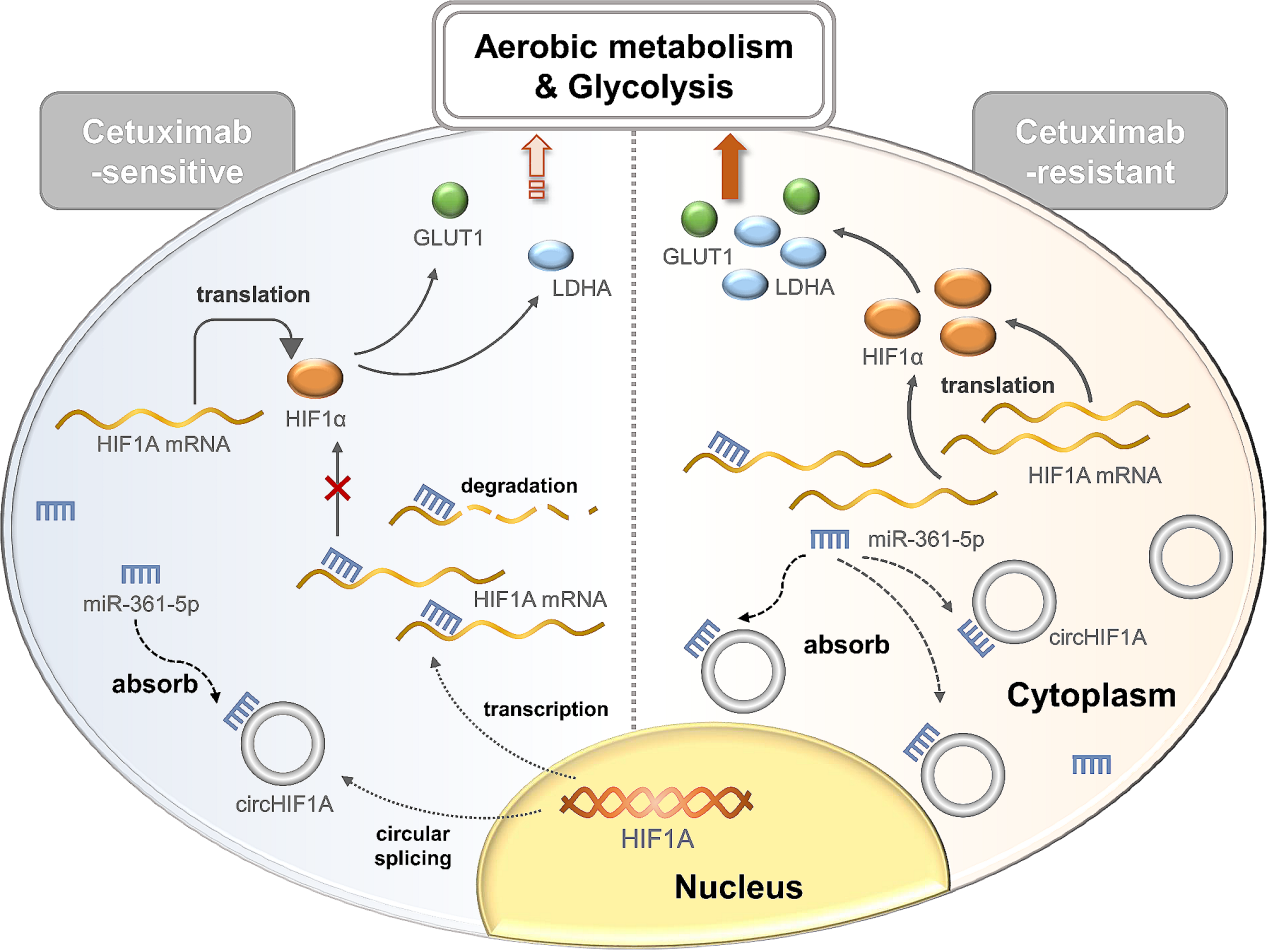
Molecular mechanism of Cetuximab resistance mediated by circHIF1A. The low expression of circHIF1A in CRC cells will release a large amount of free miR-361-5p, which negatively regulates the expression of HIF1A, leading to the decline of downstream GLUT1 and LDHA levels, the reduction of aerobic metabolism and glycolysis, as well as cell proliferation. Therefore, the response to Cetuximab treatment is increased (Cetuximab-sensitive). On the contrary, overexpression of circHIF1A in CRC cells competitively binds to miR-361-5p, resulting in a significant increase in HIF1A mRNA and HIF1α levels, promoting the expression of GLUT1 and LDHA. Then, the aerobic metabolism and glycolysis are enhanced, leading to increased proliferation and decreased response to Cetuximab treatment (Cetuximab-resistant)

The hypoxic microenvironment of tumors is closely associated with the proliferation, differentiation, angiogenesis, energy metabolism, and medicine resistance [37]. Due to the rapid proliferation of tumors, tumor cells in hypoxic regions mainly adapt to low oxygen pressure by activating certain pathways, with HIF1 being one of the most crucial factors [38–40]. HIF1 activates more than 100 downstream genes, commonly including vascular endothelial growth factor (VEGF), erythropoietin (EPO), GLUT1, and LDHA, regulating important biological processes required for tumor survival and development [41]. In this study, high level of circHIF1A competitively bound and downregulated miR-361-5p, thus relieving the negative regulation of miR-361-5p on HIF1A and increasing the level of linear HIF1A mRNA. Elevated level of HIF1α facilitates the metabolic adaptation of hypoxic tumor cells by increasing utilization and uptake of glucose, or redirecting the glucose metabolism from oxidative phosphorylation to glycolysis [42]. This metabolic transformation is mainly mediated by HIF1α though inducing overexpression of GLUT and enzymes involved in the glycolytic pathway [43]. GLUT1 is a major vector mediating cellular glucose transport and is widely present in human body. Overexpression of GLUT1 represents an overactive glycolytic phenotype. Ischemia and hypoxia during the malignant transformation lead to metabolic abnormalities, causing a significant increase in GLUT1 expression to enhance glucose transport and meet the metabolic needs of tumor cells [44]. LDHA is an important enzyme that promotes and maintains glycolysis, and the proliferation of cancer cells depends on its activity [45]. Tumor cells with inactive LDHA exhibit slower proliferation due to reduced ATP release [46]. In addition, the level of LDHA is related to chemotherapy sensitivity [47]. Inhibiting LDHA can reduce the resistance of CRC cells to 5-FU [48] and Oxaliplatin [49]. In this study, the expression levels of GLUT1, LDHA, PFKFB3, PKM2 and HK2 were elevated in Cetuximab resistant cells, and downregulating circHIF1A reduced the promoter activity and expression of GLUT1 and LDHA, suggesting that the increased level of glycometabolism, especially glycolysis, in Cetuximab resistant CRC cells is closely related to the intracellular high level of circHIF1A. The molecular mechanism of Cetuximab resistance mediated by circHIF1A in CRC is illustrated in Fig. 8.

## Conclusion

CircHIF1A is highly expressed in Cetuximab resistant CRC cells and appears to be a complementary biomarker for predicting Cetuximab efficacy in mCRC patients. The circHIF1A/miR-361-5p/HIF1A network, which affects cellular aerobic metabolism and glycolysis levels, is a potential mechanism mediating Cetuximab resistance in CRC.

### Electronic supplementary material

Below is the link to the electronic supplementary material.

Supplementary Material 1: Supplementary Fig. 1. Detection of KRAS, NRAS and BRAF in LIM1215-R: No mutations were found in KRAS (p.G12A, p.G12C, p.G12D, p.G12R, p.G12S, p.G12V, p.G13C, p.G13D), NRAS (p.G12D, p.Q61K, p.Q61R) or BRAF (p.v600E-T/A, p.v600E-TG/AA, p.v600D-TG/AT, p.v600K-GT/AA) in LIM1215-R.

Supplementary Material 2: Supplementary Fig. 2. qRT-PCR validation of differentially expressed circRNA: (A) Upregulated circRNAs in LIM1215-R. (B) Downregulated circRNAs in LIM1215-R. ^*^P < 0.05, ^***^P < 0.001.

Supplementary Material 3: Supplementary Fig. 3. Screening circRNAs influencing the sensitivity of CRC to Cetuximab: A. Validation of transfection efficiency. B-C. Changes in proliferation (B) and colony formation (C) of LIM1215-R after circRNAs downregulation (Cetuximab 5 µg/mL). D-E. Changes in proliferation (D) and colony formation (E) of LIM1215-R after circRNAs overexpression (Cetuximab 5 µg/mL). F-G. Changes in proliferation (F) and colony formation (G) of LIM1215 after hsa_circ_0007976 overexpression (Cetuximab 0 and 5 µg/mL). H-I. The proliferation (H) and colony formation (I) of LIM1215-R were not changed after hsa_circ_0007976 downregulation (Cetuximab 0 µg/mL). ^*^P < 0.05, ^**^P < 0.01, ^***^P < 0.001.

Supplementary Material 4: Supplementary Fig. 4. Detection of RNAs and protein levels in rescue experiments: A-B. The expression of circHIF1A (A) and miR-361-5p (B) were detected after circHIF1A and/or miR-361-5p knockdown. C-D. HIF1A mRNA (C) and HIF1α protein (D) levels alteration after circHIF1A and/or miR-361-5p downregulation. E-F. The expression of circHIF1A (E) and miR-361-5p (F) were detected after circHIF1A knockdown and/or HIF1A overexpression. G-H. HIF1A mRNA (G) and HIF1α protein (H) levels alteration after circHIF1A knockdown and/or HIF1A overexpression. ^*^P < 0.05, ^**^P < 0.01, ^***^P < 0.001.

Supplementary Material 5

Supplementary Material 6

## Data Availability

All the data and materials supporting the conclusion of this study have been included within the article.

## Abbreviations

EGFR: epidermal growth factor receptor
mCRC: metastatic colorectal cancer
ncRNAs: non-coding RNAs
FISH: fluorescence in situ hybridization
RIP: RNA immunoprecipitation
IHC: immunohistochemical staining
HIF1A: hypoxia-inducible factor 1 A
GLUT1: glucose transporter 1
LDHA: lactate dehydrogenase A
CRC: colorectal cancer
5-FU: 5-Fluorouracil
mAbs: monoclonal antibodies
OS: overall survival
NCCN: National Comprehensive Cancer Network
ESMO: European Society for Medical Oncology
PD: progressive disease
HER-2: human epidermal growth factor receptor-2
circRNA: circular RNA
NSCLC: non-small cell lung cancer
miRNAs: micro RNAs
lncRNAs: long non-coding RNAs
DMEM: Dulbecco’s modified Eagle medium
siRNA: small interfering RNA
shRNA: short hairpin RNA
MSS: microsatellite stability
PFS: progression-free survival
qRT-PCR: quantitative real-time polymerase chain reaction
ECL: enhanced chemiluminescence
CCK8: cell counting kit-8
PI: propidium iodide
DAPI: 4,6-diamidino-2-phenylindole
AGO2: Argonaute 2
ENCORI: Encyclopedia of RNA Interactomes
IF: immunofluorescence
EDTA: ethylene diamine tetraacetie acid
HE: hematoxylin and eosin
HR: hazard ratio
bp: base pairs
AE: adverse events
ORR: objective response rate
DCR: disease control rate
mPFS: median PFS
CI: confidence interval
MDT: multi-disciplinary treatment
LV: leucovorin
ETS: early tumor regression
DpR: deepness of response
NED: no evidence of disease
ADCC: antibody-dependent cell-mediated cytotoxicity
ceRNA: competitive endogenous RNA
VEGF: vascular endothelial growth factor
EPO: erythropoietin
