# Supplementary figures and images for: CircHIF1A induces cetuximab resistance in colorectal cancer by promoting HIF1α-mediated glycometabolism alteration
Source: Biol Direct. 2024 May 7;19:36. doi: 10.1186/s13062-024-00478-x (PMC11075259; doi:10.1186/s13062-024-00478-x)

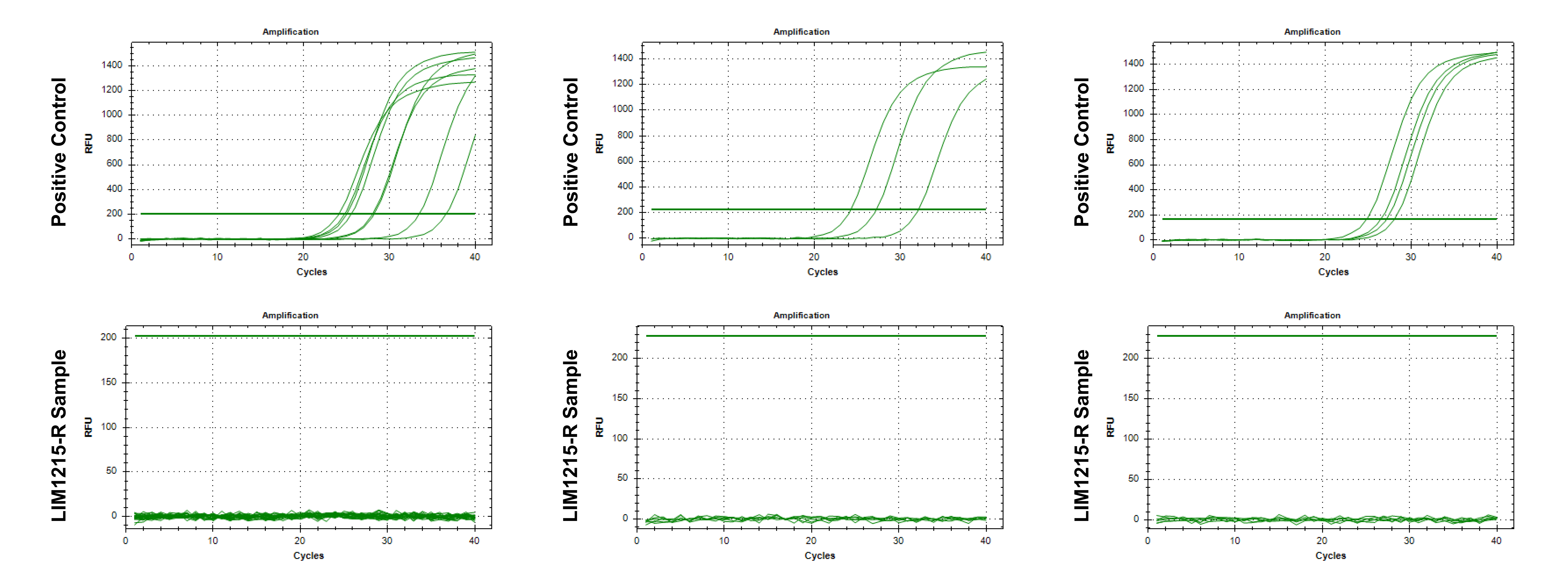

Supplement: Supplementary file 1 — Supplementary Material 1: Supplementary Fig. 1. Detection of KRAS, NRAS and BRAF in LIM1215-R: No mutations were found in KRAS (p.G12A, p.G12C, p.G12D, p.G12R, p.G12S, p.G12V, p.G13C, p.G13D), NRAS (p.G12D, p.Q61K, p.Q61R) or BRAF (p.v600E-T/A, p.v600E-TG/AA, p.v600D-TG/AT, p.v600K-GT/AA) in LIM1215-R. [file 13062_2024_478_MOESM1_ESM.png]

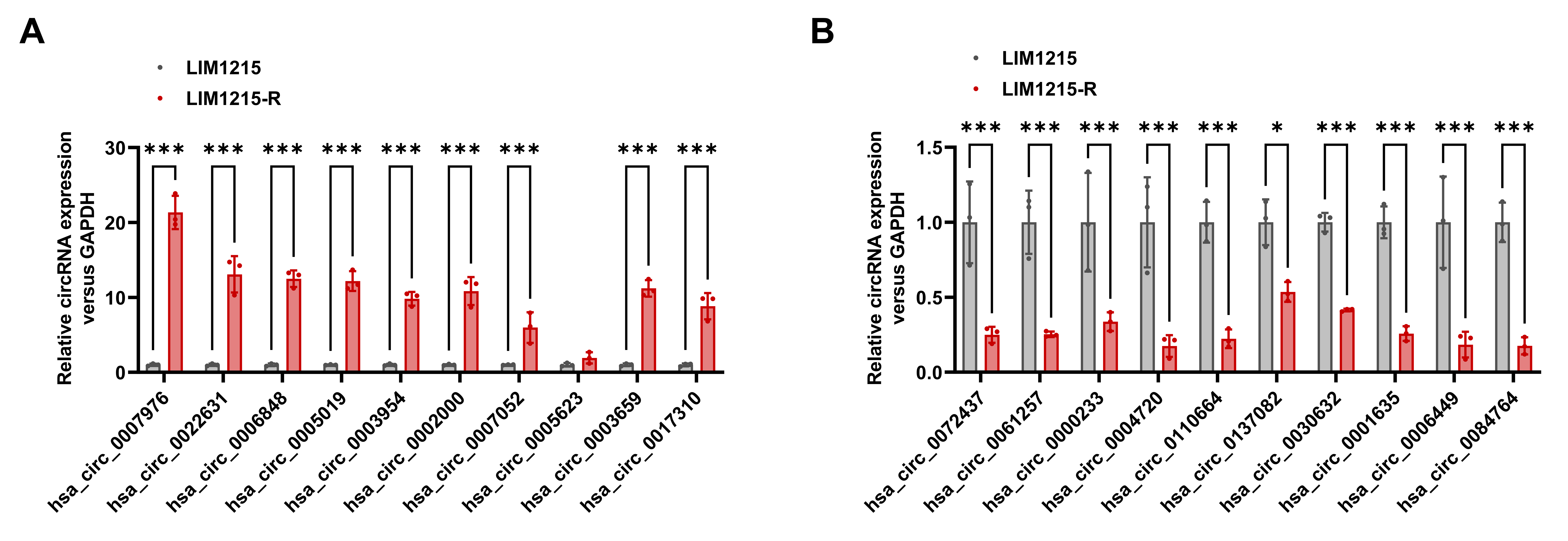

Supplement: Supplementary file 2 — Supplementary Material 2: Supplementary Fig. 2. qRT-PCR validation of differentially expressed circRNA: (A) Upregulated circRNAs in LIM1215-R. (B) Downregulated circRNAs in LIM1215-R. *P < 0.05, ***P < 0.001. [file 13062_2024_478_MOESM2_ESM.png]

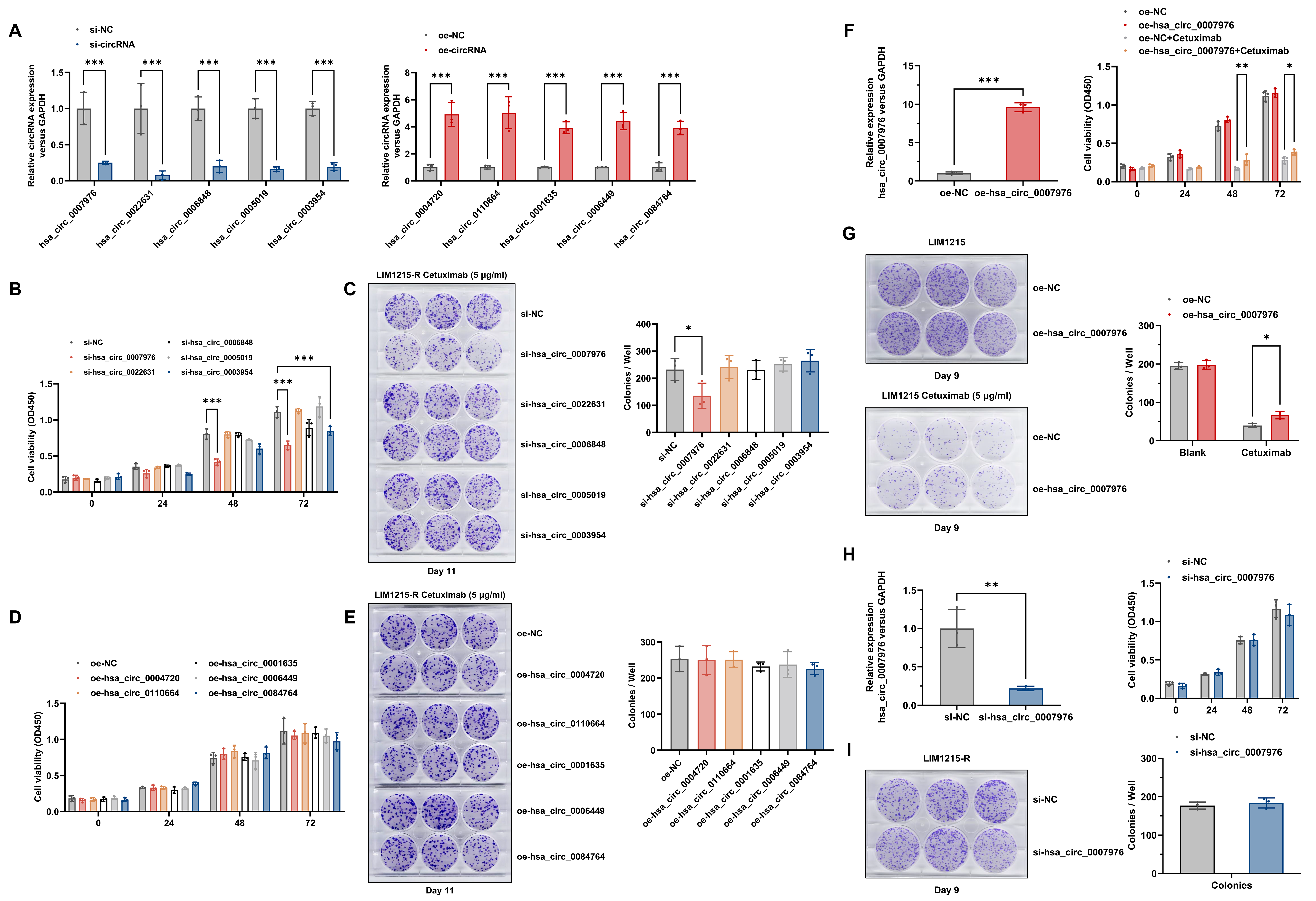

Supplement: Supplementary file 3 — Supplementary Material 3: Supplementary Fig. 3. Screening circRNAs influencing the sensitivity of CRC to Cetuximab: A. Validation of transfection efficiency. B-C. Changes in proliferation (B) and colony formation (C) of LIM1215-R after circRNAs downregulation (Cetuximab 5 µg/mL). D-E. Changes in proliferation (D) and colony formation (E) of LIM1215-R after circRNAs overexpression (Cetuximab 5 µg/mL). F-G. Changes in proliferation (F) and colony formation (G) of LIM1215 after hsa_circ_0007976 overexpression (Cetuximab 0 and 5 µg/mL). H-I. The proliferation (H) and colony formation (I) of LIM1215-R were not changed after hsa_circ_0007976 downregulation (Cetuximab 0 µg/mL). *P < 0.05, **P < 0.01, ***P < 0.001. [file 13062_2024_478_MOESM3_ESM.png]

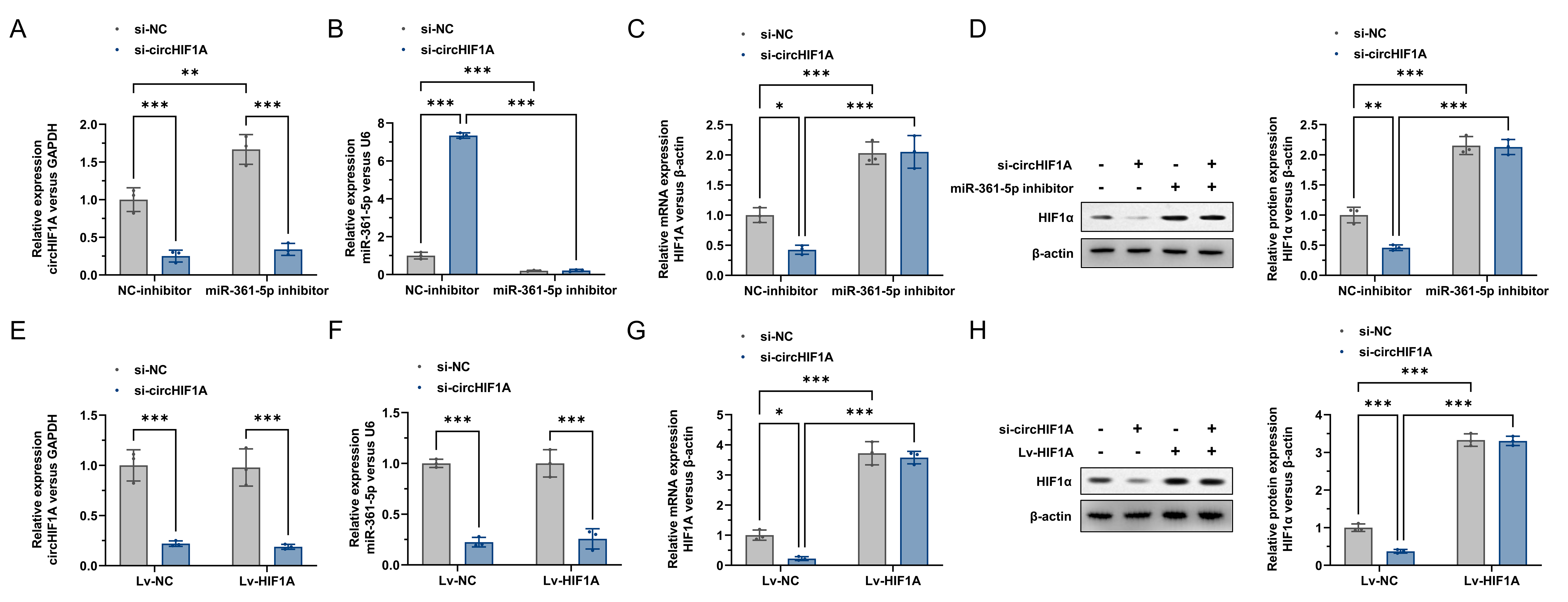

Supplement: Supplementary file 4 — Supplementary Material 4: Supplementary Fig. 4. Detection of RNAs and protein levels in rescue experiments: A-B. The expression of circHIF1A (A) and miR-361-5p (B) were detected after circHIF1A and/or miR-361-5p knockdown. C-D. HIF1A mRNA (C) and HIF1α protein (D) levels alteration after circHIF1A and/or miR-361-5p downregulation. E-F. The expression of circHIF1A (E) and miR-361-5p (F) were detected after circHIF1A knockdown and/or HIF1A overexpression. G-H. HIF1A mRNA (G) and HIF1α protein (H) levels alteration after circHIF1A knockdown and/or HIF1A overexpression. *P < 0.05, **P < 0.01, ***P < 0.001. [file 13062_2024_478_MOESM4_ESM.png]
