# Supplementary material for: CircHIF1A induces cetuximab resistance in colorectal cancer by promoting HIF1α-mediated glycometabolism alteration
Source: Biol Direct. 2024 May 7;19:36. doi: 10.1186/s13062-024-00478-x (PMC11075259; doi:10.1186/s13062-024-00478-x)
Supplement: Supplementary file 5 — Supplementary Material 5 [file 13062_2024_478_MOESM5_ESM.docx]

**Supplementary Materials and Methods**

**Quantitative real-time polymerase chain reaction (qRT-PCR)**

Total RNA was extracted using Trizol reagent (Invitrogen, CA, USA), and genomic DNA was removed. The concentration and purity of total RNA were determined using the NanoDrop 2000 microspectrophotometer. The purified total RNA was reverse transcribed into cDNA using the PrimeScript RT Reagent Kit, and the expression of circRNA, mRNA, and miRNA was determined using the SYBR Green qRT-PCR kit with stem-loop primers (Takara, Otsu, Japan). GAPDH and β-actin were used as the internal control for circRNA, mRNA and protein, and U6 was used as the internal control for miRNA. qRT-PCR was performed on an ABI viiA7 Real-Time PCR system (Applied Biosystems, Foster City, CA, USA). The relative expression levels of RNA were calculated using the 2^−ΔΔCT^ method. All primers were designed and synthesized by General Biosystems (Hefei, Anhui, China), and the primer sequences are shown in Supplementary Table 2.

**Western blotting**

LIM1215 and LIM1215-R cells were collected, and total cellular protein was extracted using RIPA lysis buffer (Beyotime). The protein concentration was measured using the BCA protein analysis kit (Beyotime). The protein samples were separated on 10% SDS-PAGE gels and then transferred to PVDF membranes (Millipore, Schwalbach, Germany). The membranes were blocked with 5% skim milk for 1h and then incubated with primary antibodies (HIF1α 1:2000, GLUT1 1:1000, LDHA 1:1500) at 4°C overnight. Subsequently, the membranes were incubated with HRP-conjugated secondary antibodies (1:5000) at room temperature for 1h, and the immunoreactive bands were detected using an enhanced chemiluminescence (ECL) kit (Pierce, Waltham, MA, USA) and analyzed using ChemiDoc MP imaging system (Bio-Rad, Hercules, CA, USA). All antibodies used are listed in Supplementary Table 3.

**Cell Counting Kit-8 (CCK8) proliferation assay**

The proliferation ability of CRC cells was detected using the CCK8 colorimetric method. Logarithmic growth phase (1×10^3^) cells were collected and seeded into 96-well plates. Subsequently, 10μl of CCK8 solution was added to each well, and the plates were incubated at 37°C for 4h. The absorbance at 450nm was measured using an automatic microplate reader (Synergy 4, BioTek, Winooski, VT, USA) to indirectly calculate the number of viable cells.

**Clone formation assay**

Preparation of 1.2% and 0.7% low-concentration agarose aqueous solutions, 1.2% agarose solution was mixed with RPMI-1640 medium at a 1:1 ratio and added to a 6 well plate (1.5ml/well). 0.7% agarose solution was mixed with RPMI-1640 medium containing fetal bovine serum, and LIM1215 and LIM1215-R cells were suspended in the mixture. Subsequently, the cells were seeded into the 6-well plate (1×10^3^ cells/well), and were incubated for 2 weeks at 37°C. The colonies were fixed with 4% polyformaldehyde and stained with 0.1% crystal violet for counting.

**Cell apoptosis and cell cycle analysis**

LIM1215 and LIM1215-R cells were fixed overnight in 70% ethanol pre-cooled at 4°C, stained with propidium iodide (PI), and analyzed using a flow cytometer (BD FACSCelesta). The cells were stained with Annexin V-FITC and PI, and the apoptotic rate was detected using a flow cytometer.

**Cell metabolism assay**

LIM1215 and LIM1215-R cells were seeded into XF96 wells and cultured in 0-, 5-, and 10-mM lactate media (1.5 × 10^4^ cells/well). Two 96-well plates were prepared using this method and incubated under normoxia and hypoxia conditions for 24h before performing cell metabolism analysis. The mitochondrial metabolism and glycolytic activity of the cells under different lactate and oxygen concentrations were determined using the XF Cell Mito Stress Test Kit (Cat# 103015-100) and the XF Glycolysis Stress Test Kit (Cat# 103020-100), respectively, following the manufacturer’s instructions. The OCR and ECAR value were measured three times under basal conditions and normalized to protein concentration per well. Statistical analysis was performed using GraphPad Prism.

**RNase R resistance analysis**

Total RNA was incubated with or without 3U/mg RNase R (Epientre Technologies, Madison, WI, USA) at 37°C for 30min. Then, specific primers were used to reverse transcribe and quantify the RNA level by quantitative RT-PCR.

**Fluorescence in situ hybridization (FISH)**

LIM1215 and LIM1215-R cells were fixed with 4% formaldehyde for 15min, washed with PBS, treated with pepsin, and dehydrated with ethanol. FISH detection was performed using the FISH kit (Sigma-Aldrich, St. Louis, MO, USA) according to the manufacturer’s instructions. The cell nuclei were stained with 4,6-diamidino-2-phenylindole (DAPI, Beyotime, China). Cy3-labeled hsa_circ_0007976 (circHIF1A) probe was observed and imaged using a confocal microscope, and the sequence was shown in Supplementary Table 4.

**RNA immunoprecipitation (RIP)**

The Imprint^®^ RNA Immunoprecipitation Kit (Sigma-Aldrich, St. Louis, MO, USA) was used for RIP. LIM1215-R cells were lysed, and protein A magnetic beads coupled with anti-Argonaute 2 (AGO2) or anti-IgG antibodies were incubated with the cell lysate overnight at 4°C. The cells were washed with buffer, and qRT-PCR was used to detect the RNA in the RNA-binding protein complex bound to the magnetic beads.

**RNA pull-down**

LIM1215-R cells (1.5×10^7^) were lysed, and biotin-labeled miRNA-361-5p probes were synthesized by General Biologicals (Hefei, Anhui, China) for incubation with streptavidin-coated agarose beads (Thermo Science). The cell lysate containing the miRNA-361-5p probes or oligonucleotide probes was incubated with the beads overnight at 4°C. The RNA complex bound to the beads was washed with wash buffer, and qRT-PCR was used to detect the abundance of circHIF1A pulled down by the miRNA-361-5p probe. The sequences of probes were shown in Supplementary Table 4.

**Luciferase reporter assay**

Using the Encyclopedia of RNA Interactomes (ENCORI) software (http://starbase.sysu.edu.cn) based on PITA, RNA22, miRMAP, microT, PicTar, TargetScan, and miRanda, potential miRNAs that bind to circHIF1A and HIF1A were analyzed. For circHIF1A and miRNA-361-5p, wild-type and mutant circHIF1A sequences with miRNA-361-5p binding site were synthesized and inserted into the psiCheck2 dual-luciferase reporter vector (Promega). Subsequently, circHIF1A-WT or circHIF1A-Mut were co-transfected with miRNA-361-5p mimics or negative controls into LIM1215-R cells. Additionally, wild-type and mutant HIF1A sequences with miRNA-361-5p binding site were synthesized, inserted into the psiCheck2 dual-luciferase reporter vector, and co-transfected with miRNA-361-5p mimics or negative controls into LIM1215-R cells for HIF1A and miRNA-361-5p. The dual-luciferase reporter assay was performed using the Dual-Luciferase Reporter Assay System (Promega) to detect luciferase activity after 48 hours.

**Immunohistochemistry (IHC) and Immunofluorescence (IF)**

Tumor tissues were cut into 4μm thick sections, deparaffinized in xylene, and rehydrated in alcohol. Antigen retrieval was performed by heating the tissue sections in ethylene diamine tetraacetie acid (EDTA) solution (100°C, 30min). After cooling, the tissue sections were treated with 0.3% hydrogen peroxide solution for 15min, washed with PBS for 5min, and blocked with 3% BSA solution at room temperature for 30min. The tissue sections were then incubated with mouse mAbs against human HIF1α (1:200), GLUT1 (1:400), and LDHA (1:400) overnight at 4°C, followed by incubation with goat anti-mouse secondary antibodies conjugated with horseradish peroxidase. The sections were stained with 3,3’-diaminobenzidine and counterstained with hematoxylin. After dehydration, clearing, and mounting, images were observed under a microscope. For IF, cells were incubated with rabbit mAbs against Ki67 (1:400) and EdU (1:200) overnight at 4°C, followed by incubation with AlexaFluor488-labeled secondary antibodies (A27034, Thermo Fisher). Images were captured using a Zeiss LSM800 fluorescence microscope (Zeiss, Jena, Germany). All antibodies used are listed in Supplementary Table 3.

**Xenograft transplantation experiment**

Five-week-old male BALB/c nude mice were randomly divided into four groups, each containing five mice. The blank control group was inoculated with LIM1215-R cells (10^7^ cells/0.15mL PBS) transfected with empty vector. The Cetuximab control group was inoculated with LIM1215-R cells transfected with empty vector and treated with Cetuximab at a dose of 1mg/kg. The experimental group was inoculated with LIM1215-R cells transfected with sh-circHIF1A. The Cetuximab experimental group was inoculated with LIM1215-R cells transfected with sh-circHIF1A and treated with Cetuximab at a dose of 1mg/kg. Tumor volume was measured using vernier calipers every 5 days, and the tumor growth curve was plotted. After 30 days of treatment, the mice were euthanized, and the tumor volume and weight were measured. The tumor sections were stained with hematoxylin and eosin (HE) and subjected to IHC staining.
