# Supplementary material for: CircHIF1A induces cetuximab resistance in colorectal cancer by promoting HIF1α-mediated glycometabolism alteration
Source: Biol Direct. 2024 May 7;19:36. doi: 10.1186/s13062-024-00478-x (PMC11075259; doi:10.1186/s13062-024-00478-x)
Supplement: Supplementary file 6 — Supplementary Material 6 [file 13062_2024_478_MOESM6_ESM.docx]

**Supplementary Table 1. Sequences of vectors**

| **Gene** | **Sequence (5’-3’)** |
| --- | --- |
| si-hsa_circ_0007976 | ACAGAAATGGTGATTTGGATA |
| sh-hsa_circ_0007976 | ACAGAAATGGTGATTTGGATATTCAAGAGATATCCAAATCACCATTTCTGTTTTTTT |
| si-hsa_circ_0022631 | [AAGAAATGCCAGCAGGTCAAT](http://blast.ncbi.nlm.nih.gov/Blast.cgi?PROGRAM=blastn&PAGE_TYPE=BlastSearch&LINK_LOC=blasthome&QUERY=%3ehsa_circ_0022631-siRNA1%0AAAGAAATGCCAGCAGGTCAAT&DATABASE=nr&EQ_MENU=Homo%C2%A0sapiens%C2%A0(taxid:9606)) |
| si-hsa_circ_0006848 | [ACCCTGGAAAGGATGACATCT](http://blast.ncbi.nlm.nih.gov/Blast.cgi?PROGRAM=blastn&PAGE_TYPE=BlastSearch&LINK_LOC=blasthome&QUERY=%3ehsa_circ_0006848-siRNA1%0AACCCTGGAAAGGATGACATCT&DATABASE=nr&EQ_MENU=Homo%C2%A0sapiens%C2%A0(taxid:9606)) |
| si-hsa_circ_0005019 | [ATGAGAACATGGTCCAAGACA](http://blast.ncbi.nlm.nih.gov/Blast.cgi?PROGRAM=blastn&PAGE_TYPE=BlastSearch&LINK_LOC=blasthome&QUERY=%3ehsa_circ_0005019-siRNA1%0AATGAGAACATGGTCCAAGACA&DATABASE=nr&EQ_MENU=Homo%C2%A0sapiens%C2%A0(taxid:9606)) |
| si-hsa_circ_0003954 | [ACCACCGACACGAGACGACTT](http://blast.ncbi.nlm.nih.gov/Blast.cgi?PROGRAM=blastn&PAGE_TYPE=BlastSearch&LINK_LOC=blasthome&QUERY=%3ehsa_circ_0003954-siRNA1%0AACCACCGACACGAGACGACTT&DATABASE=nr&EQ_MENU=Homo%C2%A0sapiens%C2%A0(taxid:9606)) |
| miRNA-361-5p mimic | UUAUCAGAAUCUCCAGGGGUAC |
| miRNA-361-5p inhibitor | GUACCCCUGGAGAUUCUGAUAA |

**Supplementary Table 2. Primer design for qRT-PCR**

| **Gene** | **Forward Sequence (5’-3’)** | **Reverse Sequence (5’-3’)** |
| --- | --- | --- |
| hsa_circ_0007976 | TGAGAGAAATGCTTACACACAGA | ACAAAACCATCCAAGGCTTTCA |
| hsa_circ_0022631 | TCTGGCAGAGCACCGAAC | ATCGATGTTACCCACGCTCA |
| hsa_circ_0006848 | CTGGCAGTGGTTGGAAGAAT | CATGGTGAGCTGTGGTAACC |
| hsa_circ_0005019 | CTCCGGGAGATGTACACCAC | TGTGTCAGAACCCTCACTTGA |
| hsa_circ_0003954 | TGGGGAACAGTTCAGCTCTT | TGGTTGGATCTTCTGTCGGA |
| hsa_circ_0002000 | GGAGAGCTCACGGTAGACAA | CAGTTCCTCAGGGCTGTAGT |
| hsa_circ_0007052 | TGACCTTGAGCATGACCAGA | AAAGGAGTGAGTGGCAGAGT |
| hsa_circ_0005623 | ATCATCGCCATCACCTTTGC | TGGGTCCAGTACTGAGAAGG |
| hsa_circ_0003659 | GACAACAACTTAGCAACTGAAGC | AGGTCCATATCTCCCTTTTCCA |
| hsa_circ_0017310 | CTCCAGAGATGAACAGGCCT | CGCTGTCATCCATTAGAGCA |
| hsa_circ_0072437 | TGTGTGGTTTGTGATGAGCC | TCTGTTCGAATTACTTCCCAAGC |
| hsa_circ_0061257  hsa_circ_0000233 | TCCAAGAAAGCGGAGACCTC  CGAACAAAGAAAGCTGTGAAAACAGT | CTCCTGCAGCACAAGGATAA  TCTGCTGCTTGGTAAGTTTGTTCT |
| hsa_circ_0004720 | ACCTGTACTTCGGCATGTGG | CAGGACTTAGGCTCCCCAAA |
| hsa_circ_0110664 | CCAGTCACCCGGAGCCTT | GCTCACTTGTCTTCAGTTGTATG |
| hsa_circ_0137082 | TGAGAGAGTGTTCATCTGGGAC | GTTGTAAAACTGCTACCCTCCC |
| hsa_circ_0030632 | AACTTCCCCATAAAAGCCAGA | TGGGTATCATCCAGTGCTTTG |
| hsa_circ_0001635 | TCCAGATAAAGGAGGTGATGAGG | TAGGAATAATATTTGGCTGGGGT |
| hsa_circ_0006449 | TTGACGCACTGGTATTTGGC | GCAAGAGAAGCTGCATTGTC |
| hsa_circ_0084764 | CCACCAATGCCACTTACCAG | GCTCCCAATTGCTGAACCTG |
| HIF1A | AAAGCAGTTCCGCAAGCCCT | GGTGGCAGTGGTAGTGGTGG |
| GLUT1 | GCCTGGACTCCATCATGGGC | GATGAGCAGGAAGCGGGGAC |
| LDHA | ATTATCACGGCTGGGGCACG | AGCAACTTGCAGTTCGGGCT |
| GAPDH | GCGGGGCTCTCCAGAACATC | TCCACCACTGACACGTTGGC |
| hsa-miR-4677-3p | TCTGTGAGACCAAAGAACTACT | TGGTGTCGTGGAGTCG |
| hsa-miR-361-5p | TTATCAGAATCTCCAGGGGTAC | TGGTGTCGTGGAGTCG |
| RT Primer for miRNA | CTCAACTGGTGTCGTGGAGTCGGCAATTCAGTTGAGTTTTTTTTTTTTTTAG | |
| U6 | CTCGCTTCGGCAGCACA | AACGCTTCACGAATTTGCGT |

**Supplementary Table 3. Antibodies for Western blotting and IHC**

| **Antibody** | **Manufacturer** | **Catalog number** |
| --- | --- | --- |
| HIF1α Antibody | CST | #3716 |
| GLUT1 Polyclonal antibody | Proteintech | 21829-1-AP |
| LDHA Polyclonal antibody | Proteintech | 21799-1-AP |
| Goat Anti-Rat IgG | CST | #98164 |
| GAPDH (D16H11) XP® Rabbit mAb (HRP Conjugate) | CST | #8884 |

**Supplementary Table 4. RNA probes for FISH and RNA pull-down**

| **Gene** | **probe sequence** |
| --- | --- |
| hsa_circ_0007976 | 5’-Cy3-AUCUUCAAUAUCCAAAUCACCAUUUCUGUGUGUAAGCAUU-3’ |
| miRNA-361-5p | 5’-Biotin-GUACCCCUGGAGAUUCUGAUAA-3’ |
| NC | 5’-Biotin-CCCGAACAACAACGUGUCACCA-3’ |
